# T cell infiltration and upregulation of MHCII in microglia leads to accelerated neuronal loss in an α-synuclein rat model of Parkinson’s disease

**DOI:** 10.1186/s12974-020-01911-4

**Published:** 2020-08-15

**Authors:** Meena S. Subbarayan, Charles Hudson, Lauren D. Moss, Kevin R. Nash, Paula C. Bickford

**Affiliations:** 1 Department of Molecular Pharmacology and Physiology, Morsani College of Medicine, University of South Florida, 12901 Bruce B Downs Blvd, Tampa, FL-33612 USA; 2 Center for Excellence in Aging and Brain Repair, Department of Neurosurgery and Brain Repair, Morsani College of Medicine, University of South Florida, 12901 Bruce B Downs Blvd, Tampa, FL-33612 USA; 3 Research Service, James A Haley Veterans Hospital, 13000 Bruce B Downs Blvd, Tampa, FL-33612 USA

**Keywords:** Parkinson’s disease, α-synuclein, T cells, Dopamine, Microglia, Neuroinflammation, Neurodegenerative disorders

## Abstract

**Background:**

Parkinson’s disease (PD) is the second most prevalent movement disorder characterized by up to 80% loss of dopamine (DA) neurons and accumulation of Lewy body deposits composed of α-synuclein (α-syn). Accumulation of α-syn is associated with microglial activation, leading to a pro-inflammatory environment linked with the pathogenesis of PD. Along with microglia, CD4 and CD8 T cells are observed in SNpc. The contribution of T-cells to PD development remains unclear with studies demonstrating that they may mediate neurodegeneration or act in a neuroprotective manner.

**Methods:**

Here, we assessed the contribution of T cells to PD neurodegeneration using an adeno-associated virus (AAV) coding human wild-type α-syn or GFP injected into the substantia nigra pars compacta (SNpc) in T cell deficient (athymic nude) and T cell competent (heterozygous) rats. The rats were behaviorally assessed with cylinder test to test paw bias. Following behavior testing, brains were collected and analyzed for markers of dopamine neuron, microglial activation, T cells, and α-syn expression.

**Results:**

Injection of AAV9-α-syn unilaterally into the SN of T cell competent rats resulted in a significant paw bias in comparison to the controls at 60 days post-injection. Conversely, T cell-deficient rats injected with AAV9-α-syn showed no deficit in paw bias. As expected, injected T cell competent rats demonstrated a significant increase in microglial activation (MHCII staining) as well as significant dopaminergic neuron loss. In contrast, the T cell-deficient counterparts did not show a significant increase in microglial activation or significant neuron loss compared to the control animals. We also observed CD4 and CD8 T cells in SNpc following microglial MHCII expression and dopaminergic neuron loss. The time course of T cell entry correlates with upregulation of MHCII and the peak loss of TH+ cells in the SNpc.

**Conclusion:**

These data demonstrate that T cell infiltration and microglial upregulation of MHCII are involved in α-synuclein-mediated DA neuron loss in this rat model of PD.

## Background

Parkinson’s disease (PD) is the second most prevalent neurodegenerative movement disorder affecting about 60,000 people per year in the USA with over 10 million people affected worldwide [1]. PD is characterized by motor symptoms which include tremor, postural instability, slowness of movement, and rigidity. The major hallmark of PD pathogenesis is the loss of dopamine (DA) neurons in the substantia nigra pars compacta (SNpc) resulting in dopamine depletion in the striatum where these dopaminergic neurons project [2–5]. Accumulation of α-synuclein (α-syn) oligomers is also considered one of the underlying reasons for the development and progression of PD irrespective of both familial and sporadic PD [6, 7]. An autosomal dominant familial PD is caused due to a missense mutation in the SNCA gene coding for α-syn. Gene triplication has also been shown to cause PD, indicating that aggregation of α-syn protein is associated with dopaminergic neuron loss [8–10]. Intracellular inclusions of oligomeric α-syn (Lewy body) are accompanied by surveying microglia that shift into a pro-inflammatory and phagocytic state resulting in the release of pro-inflammatory cytokines such as IL-1, IL-6, TNF-α, and CD40 ligand [11–14]. Neuronal loss in SNpc is caused, in part, from persistent inflammation in the brain resulting from pro-inflammatory microglia and dendritic cells. A prolonged pro-inflammatory state may lead to neuronal damage. Although the role of microglia has been extensively studied in various neurodegenerative disorders [11, 12, 15–18], the underlying cause for dopamine neuron loss and aggregation of α-syn in PD is still debated.

Although the relationship between microglia and neuroinflammation is extensively studied, the role of the adaptive immune system in neuroinflammation remains less established. The central nervous system (CNS) had been considered immune restricted due to the blood-brain barrier (BBB) that tightly regulates the entry of T cells, B cells, monocytes, and prevents toxins and other infectious organisms from entering; however, recent reports of the lymphatic system and CNS lymph vessels suggest that communication with the peripheral immune system is not only possible but an active process [19]. During a chronic neuro-inflammatory state, several pro-inflammatory cytokines may increase the expression of cell adhesion molecules on the endothelial cells of the BBB resulting in the migration of the peripheral immune cells to CNS [17, 19]. Post-mortem human PD brains have shown the presence of both CD4+ and CD8+ T cells in the area of damage [20]. However, the role of CD4+ and CD8+ T cells in neuroinflammation is still unclear. Recent reports have shown that infiltrating T cells in the CNS are involved either in neurodegeneration in diseases such as multiple sclerosis [19] or in being neuroprotective in certain Alzheimer’s disease model [21, 22]. In toxin-induced PD models such as MPTP and 6-hydroxy dopamine (6-OHDA), the resultant neural damage was reduced in T cell-deficient mice suggesting an active role for T cells in this process [20]. Also, α-syn injection into MHC II deficient mice did not result in microglial activation and degeneration of DA neurons [23]. This is likely due to the absence of antigen presentation by microglia to T cells. In contrast, Tregs (CD4 + CD25 + Foxp3) are shown to be neuroprotective against α-synuclein following vaccination [18]. Though most of the studies pertaining adaptive immune system and pathogenesis of PD have been done in neurotoxic models of PD such as MPTP, the interplay between T cells, microglia, and α-syn needs to be further explored. Also, the varying role of T cell subtypes and its specific relationship to the innate immune system in CNS being neuroprotective or neurotoxic needs to be investigated in PD pathogenesis.

In our study, we examined the role of microglia and T cells in an AAV-α-syn rat model of PD in T cell-deficient (athymic nude) and T cell competent (heterozygous nude) rats. Our PD model pathologically mimics the disease in the brain by aggregation of α-syn resulting in dopaminergic neuron loss in SNpc [24]. Nude rats (T cell deficient) are athymic, so the resulting T cells are not mature to its specific subtypes. Conversely, heterozygous nude rats have a thymus and are not immunodeficient like nude rats but have a substantially reduced number of CD3+ T cells when compared to F344 rats [25]. We demonstrate that in the absence of T cells, α-syn fails to upregulate MHCII in microglia, and thus there is no subsequent DA neuron loss. Furthermore, our data suggests that T cells are necessary for neurodegeneration in SNpc and that antigen presentation by microglia using MHCII to T cells is a critical step in the neurotoxic process of DA neurons in response to α-syn. Likewise, we observed a progressive loss of dopamine neurons coinciding with a gradual increase in activated microglia expressing MHCII, CD4, and CD8 T cells in SNpc indicating a strong correlation between microglial MHCII expression and infiltration of T cells in SNpc during damage.

## Methods

### Viral vectors and animal husbandry

All animal experiments were conducted in accordance with the National Institute of Health Guide and Use of Laboratory Animals and were approved by the Institutional Animal Care and Use Committee of the University of South Florida. Three-month-old male nude rats (NIH-Foxn1^rnu^, Charles River), male heterozygous nude rats (Foxn1^rnu^/Foxn1^+^, Charles River), and male Fisher 344 rats were pair-housed in environmentally controlled conditions (12:12 h day: night cycle at 21 ± 1 °C) and they were provided with food and water ad libitum. Ten animals per group were used (nude rats—GFP, α-synuclein; heterozygous nude rats—GFP, α-synuclein). Human wild-type α-synuclein or green fluorescent protein (GFP) expressing recombinant adeno-associated virus serotype 9 was produced with CBA promoter according to the protocol described in Nash et al. [26]. Animals were injected unilaterally in the right substantia nigra (ipsilateral) with 2 μl of the rAAV serotype 9 expressing either human wild-type α-synuclein (~ 1 × 10^13^ vg/ml) or GFP (~ 0.6 × 10^13^ vg/ml) at a flow rate of 2.5 μl/min. Convection enhanced delivery (CED) method of delivery was followed for the viral injection [27]. Stereotactic surgery was performed with the following injection coordinates for the delivery of rAAV; lateral: −2.00 mm, anteroposterior: −5.2 mm, and dorsoventral: −8.2 mm from bregma.

### Behavioral testing—cylinder test

The forelimb activity of the rats was tested using a cylinder behavioral test. Animals were placed in a cylinder of 24 cm height and 16 cm in diameter. The first twenty-forelimb contacts to the wall of the cylinder were recorded for each animal while rearing. The test was carried out before surgery, 1-month, and 2-month post-surgery by blinded observers. The percentage of left versus right or both paw touches was calculated.

### Immuno-histochemical and immunofluorescence analysis

After day 15 (2 weeks), 30 (4 weeks), 45 (6 weeks), and 60 (8 weeks), the rats were anesthetized and transcardially perfused with 0.1 M phosphate-buffered saline at pH 7.2 (PBS) followed by 4% paraformaldehyde (PFA) in PBS. Brain and spleen were removed and fixed in 4% PFA/PBS overnight. They were then transferred to 30% sucrose in PBS for at least 16 h until the brains equilibrated, at which point they were sectioned coronally at 40 μm using cryostat and the sections were stored at −20 °C in a cryoprotectant liquid for further processing. Immunostaining was performed on every sixth free-floating section spanning the substantia nigra of the brain or spleen for each animal. In order to block the endogenous peroxidase activity, the sections were either treated with tris buffered saline with sodium periodate (NaIO_4_) for tyrosine hydroxylase (TH) staining or 40% methanol/2% H_2_O_2_ (hydrogen peroxide) in 0.1 M PBS buffer for NeuN, Iba1, and OX-6 staining for 20 min at room temperature (RT, 60 rpm). For CD4, CD8 T cell, α-syn211, and syn33 staining, the free-floating sections were treated with 1X SSC buffer (Sigma; S6639-1 L) for 40 min at 80 °C followed by incubation with 5% H_2_O_2_ for 20 min at RT (60 rpm). The tissue sections were washed with 0.1 M PBS. Followed by washing, the tissues were blocked with a blocking buffer (PBS/0.3% Triton X-100/10% horse serum or goat serum) for 60 min at RT (60 rpm). Tissues were then incubated with primary antibody (mouse anti-TH (1:10000), Immunostar (Cat no: 22941); mouse anti-NeuN (1:1000), Millipore (Cat no: MAB 377B); rabbit anti-rat Iba1 (1:2000), Wako (Cat no: 019-19741); mouse anti-RT1B (OX-6) (1:750), BD Biosciences (Cat no: 554926); mouse anti-CD4 (1:150), BioRad (Cat no: MCA55G); mouse anti-CD8 (1:200), Abcam (Cat no: ab33786 ); mouse anti-α-syn211 (1:10000), Abcam (Cat no: ab80627); rabbit anti human-α-syn33 (1:500), Dr. Rakez Kayed [28]) made in PBS containing 3% horse serum or 3% goat serum (for Iba1, syn33), 0.1% Triton X-100 overnight at 4 °C (60 rpm). The following day, sections were washed of in PBS containing 3% horse serum or 3% goat serum (for Iba1, syn33) and incubated with horse anti-mouse or goat anti-rabbit (for Iba1, syn33) secondary antibody at a concentration of 1:1000 in PBS/Triton X-100/serum solution for 60 min at RT (60 rpm). The secondary antibody was amplified by incubating the tissues with avidin-biotin substrate (ABC kit, Vector Labs (Cat# PK-6100)) for 60 min at RT (60 rpm). As final step, the tissues were developed using 3, 3′—Diaminobenzidine tetra-hydrochloride (DAB (Sigma, Cat no: D4418)) for TH, NeuN, CD4, and CD8 staining and with metal enhancer (nickel, Ni (Sigma, Cat no: D0426)) for OX-6 (MHCII), α-syn211, Iba1, and syn33 staining. Free-floating tissues were then mounted onto a glass slide and dried overnight. The following day, the slides were dehydrated and cover-slipped using DPX mounting medium. For immunofluorescence staining, the tissues were incubated with secondary antibodies, goat-anti-mouse Alexa Fluor 488 (for MHCII; 1:500), and goat anti-rabbit Alexa Fluor 594 (for Iba1; 1:500) for 60 min at RT (60 rpm) and mounted onto slides cover-slipped with hard-set DAPI (Vector Labs). The spleen tissues were stained as a positive control for CD4 and CD8 T cell staining (Supplemental Figure 1).

### Cell culture, treatment, and ELISA

Primary microglia were obtained from young (3 months) nude and heterozygous nude rats. Rats were euthanized with CO_2_ according to the approved IACUC protocol. The rats were perfused with PBS and the brains were dropped in ice-cold 1XHBSS (GIBO, Cat no: 14185-052) w/o Ca++ and Mg++. The brain was dissociated to small pieces using a scalpel or razor blade in a petri dish and then transferred to a 15-ml tube and centrifuged at 200 g for 2 min. The tissue was enzymatically digested using the Miltenyi Biotec’s neural dissociation kit (Cat no: 130-093-231) to obtain a single-cell suspension according to manufacture protocol. Primary microglia were isolated using Miltenyi Biotec’s LS magnetic columns and CD11b (Miltenyi Biotec, Cat no: 130-090-320) magnetic beads. The procedure yielded around 1.9-2.3 × 10^6^ cells with > 95% purity for both nude and heterozygous nude rats [29].

For LPS and TNF-α treatment, a 6-well plate was seeded with 300,000 microglial cells/well. The cells were treated with LPS (1 ng/ml and 10 ng/ml; Thermo Fisher, Cat no: O55:B5) and TNF-α (50 ng/ml and 100 ng/ml; Sigma, Cat no: T5944) in triplicate for each biological replicate at the given concentration 72 h after the cells were isolated and plated. The cells for RNA isolation were collected 3 h post-TNF-α treatment and 6 h post LPS treatment. The media isolated from the LPS-treated cells were used for ELISA testing (TNF-α Duo Set, R&D systems, Cat no: DY510-05) for TNF-α secretion as described by the manufacturer.

### RNA isolation and real-time polymerase chain reaction (RT-PCR)

RNA from LPS and TNF-α treated primary microglial cultures were isolated using the Qiagen isolation kit (Cat no: 80004) and performed according to the manufacture’s protocol. The RNA concentrations were measured using Nanodrop. The isolated RNA was converted to cDNA using high capacity RNA to cDNA kit (Applied Biosystems, Cat no: 4387406) and manufacturer protocol was followed. The primers used were TNF-α, IL1β, IL-6 (IDT Primers, ref sequence # NM_012675, NM_031512, and NM_012589) and MHCII (RT1u.D). The MHCII primer was designed for RT1u.D alpha chain mRNA 3′ end (PubMed ID: M15562) with forward and reverse primers as follows: 5′ AGACAGTGTTTCTCCCAAGG 3′; 5′GTGATCCACCTCACAGTCATAG 3′. For the RT-PCR, 1 μl of primer yielding final concentration to 500 nM (TNF-α, IL-6, IL1β, MHCII), 5 μl of SYBR Green master mix (Applied Biosystems, Cat no: A25742), and 10 ng of cDNA were mixed together in a sterile PCR tube. The RT-PCR procedure has four stages: two holding stages for 2 min each at 50 °C followed by 95 °C, cycling stage repeating for 40 cycles at 95 °C for 3 s and 60 °C for 30 s, and the last melting curve stage at 95 °C for 15 s followed by 60 °C for 1 min and again at 95 °C for 15 s (StepOnePlus Real-Time PCR system, ThermoFisher Scientific). The amplification curve and the ΔC_T_ were calculated using the Step One software. Each experimental condition was run in triplicate for each donor rat, and the RNA from each well assayed in duplicate.

### Image analysis and quantification

The tissues stained with TH, NeuN, OX-6, Iba1, α-syn33, and α-syn211 were scanned using Zeiss Mirax image scanner. The scanned images were analyzed using NearCYTE image analysis software (nearcyte.org). Using the NearCYTE image analysis software, region of interest (ROI) were imposed onto the images and was selected for the substantia nigra pars compacta (SNpc). The ROI was compared to the threshold intensity setup by an experimenter blind to the treatment groups. The comparison creates a ratio of the population of the staining in each ROI [26]. The neurodegeneration is estimated by comparing the area ratio generated for contralateral and ipsilateral side of each brain slice per animal. Our lab has previously demonstrated that data collected with the NearCYTE software accurately reflects the cell counts using the Stereo investigator software (MBF Bioscience) [26]. The TH + ve cells, α-syn oligomers stained with syn33 antibody, CD4 and CD8 T cells in the SN and spleen were also counted in every 6th section using the optical fractionator method of unbiased stereology utilizing the Stereo Investigator software (MicroBrightField) on a Nikon Eclipse 600 microscope. A grid size of 150 × 150 (for CD4 and CD8)/300 × 300 (for syn33)/140 × 140 (TH) and counting frame of 100 × 100 (syn33, CD4 & CD8)/70 × 70 (TH) [30] was employed for each animal. The ROI was outlined using a 10 × 0.45 objective and the positive cells were counted using a 40 × 0.95 objective. For immunofluorescence staining, the images were taken using the Keyence microscope and the representative images are from a single Z stack plane.

### Statistical analysis

All the bar graphs in the figures are represented as mean ± standard error of the mean. Statistical analysis was performed using the GraphPad Prism software. The timepoint interaction and treatment interaction for cylinder test was analyzed using the two-way ANOVA repeated measure with Tukey’s multiple comparison test. The treatment interaction for immunohistochemistry data were analyzed using the two-way ANOVA with Tukey’s multiple comparison test. The CD4 and CD8 T cell staining data was analyzed using the one-way ANOVA with Tukey’s multiple comparison test. The group interactions for RT-PCR data was analyzed using the two-way ANOVA with Sidak’s multiple comparison test. All significant results represented show a *p* value less than 0.05 unless otherwise mentioned.

## Results

### T cell deficient rats do not show development of paw bias

**Fig. 1:**
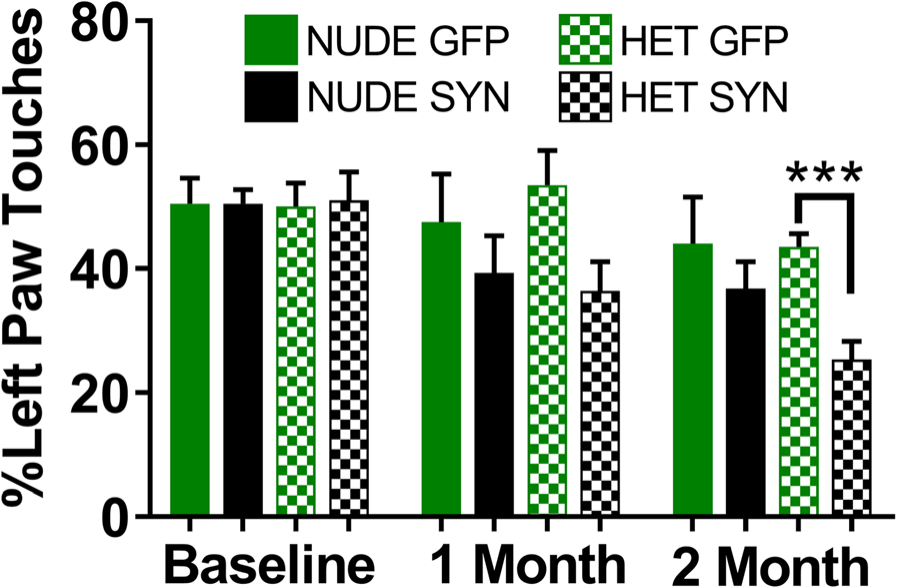
Bar graph showing results from the cylinder test for paw bias at three different time points: baseline (before surgery), 1-month post-surgery, and 2-month post-surgery. Heterozygous nude rats injected with AAV9-αsyn slowly developed a paw bias with more right paw touches that became significant at 2 months post-surgery when compared to GFP-injected controls (Two-way ANOVA, mixed-effects analysis *p* < 0.0001; *F* (1.96, 79.15) = 13.31 post hoc analysis: Tukey’s multiple comparison test, *p* = 0.0003), whereas T cell deficient nude rats injected with AAV9-αsyn did not develop a significant paw bias at any time point tested. Ten animals per group were used (nude rats—GFP, α-synuclein; heterozygous nude rats—GFP, α-synuclein). The bars in the graph represent mean±standard error of the mean

In order to understand the functional impact of the synucleinopathy in SNpc, we behaviorally assessed forelimb akinesia by performing the cylinder test on T cell deficient (nude) and T cell competent (heterozygous) rats injected unilaterally with rAAV9 expressing either human wild-type α-syn or GFP at three different time points: before surgery, 30 days (1 month) post-surgery, and 60 days (2 month) post-surgery. The nude and heterozygous nude rats used in this study were from same littermates. The nude rats injected with either AAV9-α-syn or -GFP did not show any paw preference bias at any of these time points. However, the heterozygous nude rats injected with human α-syn showed a preference for ipsilateral paw touches (Two-way ANOVA: *F* (1.96, 79.15) = 13.31, *p* < 0.05; Tukey’s multiple comparison, *p* = 0.0003) at the 60 days (2 month) post-surgery time point as expected in this model (Fig. 1). These results show that absence of T cells protects the rats from the functional impact of synucleinopathy in SNpc at 60 days (2 month) post-injection.

### Absence of T cells prevents dopaminergic neuron loss in SNpc

**Fig. 2:**
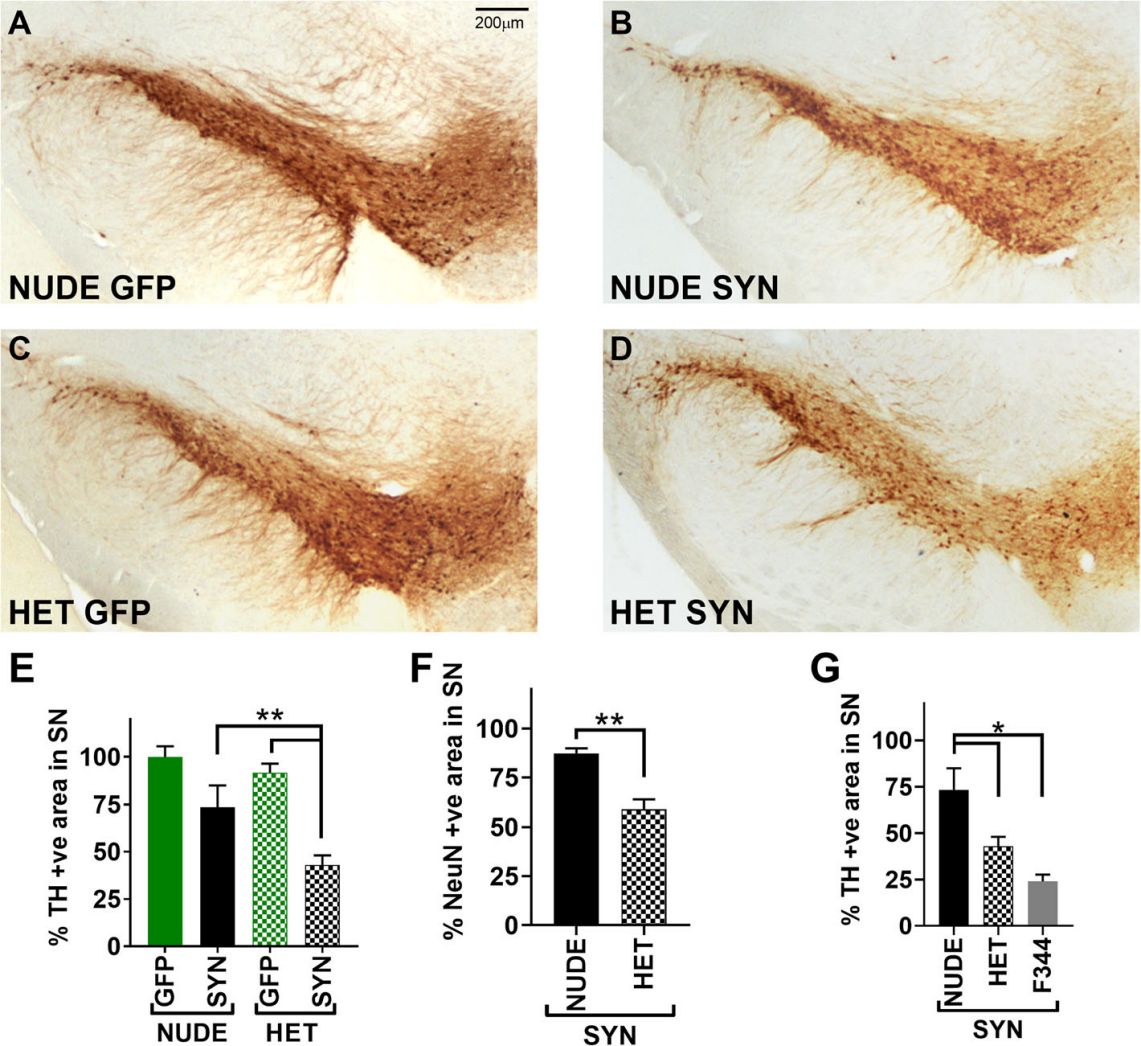
**a**-**d** Representative photomicrographs of TH (tyrosine hydroxylase—marker for dopamine neurons) staining in the substantia nigra (SN) region. **a** T cell deficient (nude) rats injected with AAV9-GFP (*n* = 10), **b** T cell deficient rats injected with AA9-human α-synuclein (αsyn) (*n* = 10), **c** T cell competent (heterozygous nude) rats injected with AAV9-GFP (*n* = 10), **d** T cell competent rats injected with AAV9-human α-synuclein (*n* = 10). **e** Bar graph shows the percentage area of positive cells in SNpc stained with TH by comparing the injected ipsilateral to the untreated contralateral side. A significant difference (two-way ANOVA *p* < 0.05; treatment: *F* (1, 32) = 23.12, *p* < 0.01; genetic background: *F* (1, 32) = 6.199, *p* < 0.05; post hoc analysis: Tukey’s multiple comparison test) of percentage positive cells was observed between the heterozygous nude rats injected with αsyn when compared to GFP injected controls. No significant difference was observed between the nude rats injected with αsyn compared to GFP. **f** Bar graph shows the percentage area of positive cells in SNpc stained with NeuN by comparing the injected ipsilateral to the untreated contralateral side for nude (*n* = 5) and heterozygous nude rats (*n* = 5) injected with α-syn. A significant difference (unpaired *t* test; *p* = 0.0026) in the percentage of NeuN+ cells was observed between the two groups. **g** Bar graph shows the percentage area of positive cells in SNpc stained with TH by comparing the injected ipsilateral to the untreated contralateral side for Fisher 344 (*n* = 10), heterozygous nude (*n* = 10), and nude rats (*n* = 10) injected with α-syn. A significant difference (one-way ANOVA *p* < 0.05; *F* (2, 24) = 10.02; post hoc analysis: Tukey’s multiple comparison test) in the percentage of TH+ cells was observed between groups. The bars in the graph represent mean ± standard error of the mean

In our previous studies, we have shown that overexpression of human α-syn using AAV9 results in dopaminergic neuronal loss in SNpc of the F344 rats [26, 30, 31]. The nude and heterozygous nude rat brains were immuno-stained for tyrosine hydroxylase (TH) 2 months after injection of AAV9-α-syn (Fig. 2a-d). TH is the rate limiting enzyme that converts tyrosine to L-DOPA (L-3, 4-dihydroxyphenylalanine), a precursor to dopamine and thus a marker of DA neurons. In GFP treated rats there was no difference in TH+ staining between the ipsilateral and contralateral substantia nigra pars compacta (SNpc). In heterozygous nude rats injected with AAV9-α-syn the percent positive area of staining for TH ratioed to the contralateral side showed significant reductions (Two-way ANOVA *F* (1, 32) = 23.12, *p* < 0.05; Tukey’s multiple comparison, *p* = 0.044) (Fig. 2e) when compared to the GFP injected controls [26]. In contrast, nude rats injected with AAV9-α-syn did not show a significant reduction in TH immunostaining. The unbiased stereological cell counts for TH for each treatment group showed the same effect as the NearCYTE area sampling method (Supplemental Figure 2). Additionally, Fischer 344 rats that have a full complement of T cells showed an even larger reduction in TH staining (Two-way ANOVA *F* (2, 24) = 10.02, *p* < 0.05; Tukey’ multiple comparison, *p* = 0.0005) (Fig. 2g) when compared to the nude rats that are deficient in T cells, possibly indicating that there may be a correlative relationship between the number of T cells and dopamine neuron loss in SNpc. Further, we stained for NeuN to determine if loss of TH was accompanied by neuronal loss in the SNpc. Like TH staining, heterozygous nude rats injected with AAV9-α-syn showed significant reduction (unpaired *t* test, *p* = 0.0026) in neurons in SNpc when compared to the nude rats injected with AAV9-α-syn (Fig. 2f). These results indicate that T cell competent rats show significant dopamine neuronal loss in SNpc with α-syn expression when compared to the T cell deficient rats, demonstrating that T cells may play a major role in dopamine neuronal loss in SNpc.

### Microglial MHCII expression is not increased in T cell deficient rats in the presence of α-synuclein

**Fig. 3:**
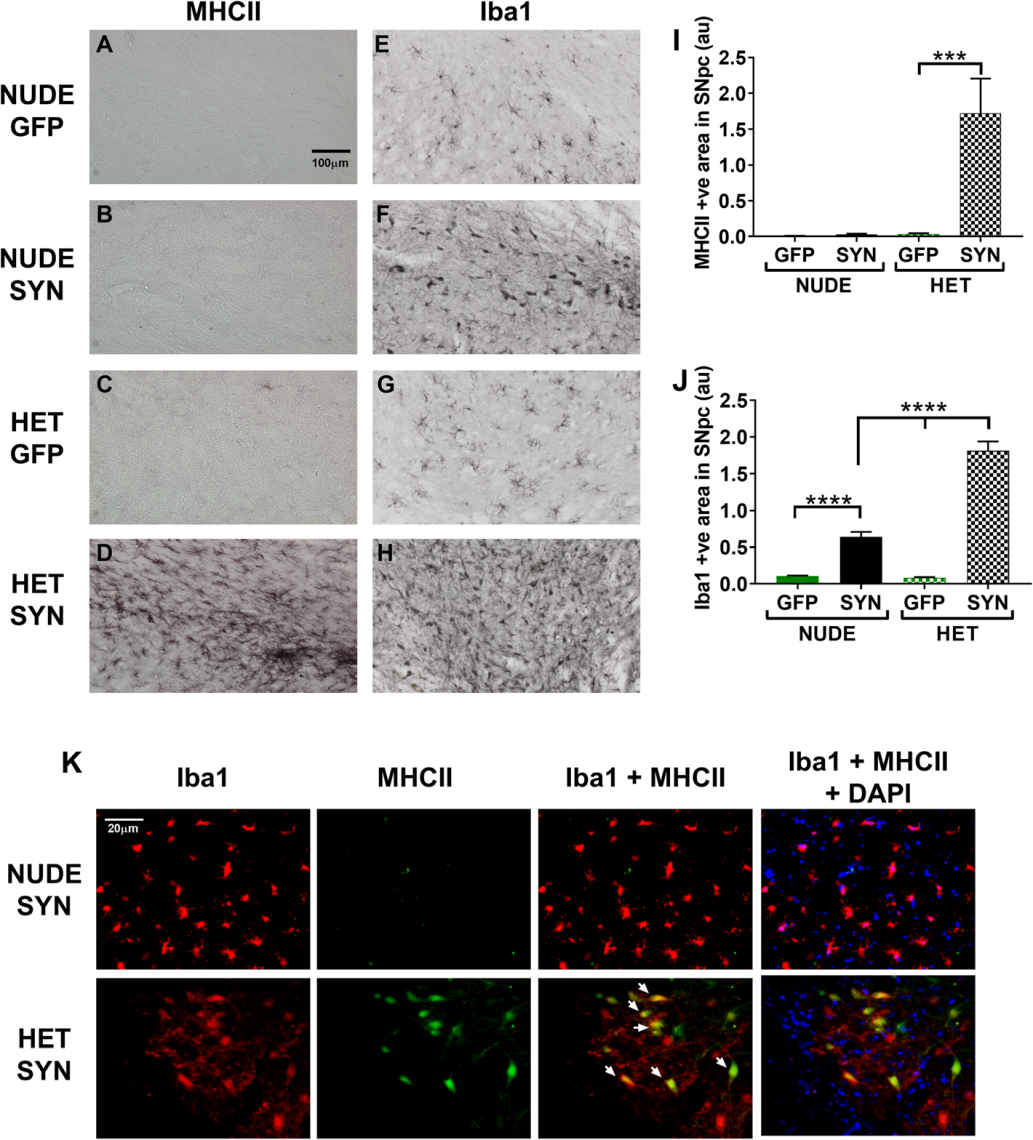
**a**-**d** Representative photomicrographs of MHCII staining for activated microglia in the SN region. **e**-**h** Representative photomicrographs of Iba1 staining for microglia in the SN region. **a**, **e** T cell deficient (nude) rats injected with AAV9-GFP (*n* = 10); **b**, **f** T cell deficient rats injected with AA9-human α-synuclein (αsyn) (*n* = 10); **c**, **g** T cell competent (heterozygous nude) rats injected with AAV9-GFP (*n* = 10); **d**, **h** T cell competent rats injected with AAV9-human α-synuclein (*n* = 10). **i** Bar graph shows the area units for MHC II staining for activated microglia in nude and heterozygous nude rats injected with either AAV9-GFP or AAV9-αsyn. The microglia get activated only in the heterozygous nude rats injected with AAV9-αsyn in SNpc region (Two-way ANOVA *p* < 0.001; treatment: *F* (1, 33) = 13.68, *p* < 0.001; genetic background: *F* (1, 33) = 13.48, *p* < 0.001; post hoc analysis: Tukey’s multiple comparison test, *p* = 0.0001). Both AAV9-αsyn and AAV9-GFP injected nude rats as well as heterozygous nude rats injected with AAV9-GFP do not show an increase in microglial activation 2-month post stereotactic surgery. **j** Bar graph shows the area units for Iba1 staining for microglia in nude and heterozygous nude rats injected with either AAV9-GFP or AAV9-αsyn in the SNpc region. Both nude and heterozygous nude rats injected with AAV9-αsyn showed increased number of Iba1 cells in SN region when compared to GFP injected controls. A significant difference was observed between nude and heterozygous nude rats injected with AAV9-αsyn (Two-way ANOVA *p* < 0.0001; treatment: *F* (1, 34) = 257.8, *p* < 0.0001; genetic background: *F* (1, 34) = 66.41, *p* < 0.0001; post hoc analysis: Tukey’s multiple comparison test). The bars in the graph represent mean ± standard error of the mean. **k** Representative photomicrographs of Iba1 and MHCII immunofluorescence staining microglia of nude and heterozygous nude rats injected with AAV9-αsyn in the SN region. The heterozygous nude rats injected with AAV9-αsyn showed activated microglia, whereas the microglia in nude rat counterparts did not have any activated microglia expressing MHCII. The activated microglia expressing MHCII are indicated with arrows

To further understand the role of microglia in dopaminergic neuron loss, we stained the SNpc for MHCII (OX6 antibody) (Fig. 3a-d, k) and Iba1 (Fig. 3e-f, k). A similar number of Iba-1 microglia are observed in the control GFP-treated nude and heterozygous nude rats (Fig. 3e, g, j). In response to AAV9-α-syn, both nude and heterozygous nude rats show morphological changes where the cell body becomes swollen (Fig. 3f, h) that when quantified shows an increased area of staining compared the GFP-treated rats of the same genotype (Two-way ANNOVA *F* (1, 34) = 257.8, *p* < 0.05; Tukey’s multiple comparison test, *p* < 0.0001) (Fig. 3j). Moreover, when comparing the microglial Iba-1 response to α-syn in the nude versus heterozygous nude rats there was a significantly higher increase in the heterozygous nude rats that may also reflect microgliosis or recruitment of additional microglia (monocytes) to SNpc (Fig. 3f, h, j) (Two-way ANNOVA; Tukey’s multiple comparison, *p* < 0.0001). Further, we measured upregulation of MHCII by immunostaining (OX-6 antibody). The number of MHCII positive microglia was only increased in heterozygous nude rats injected with AAV9-α-syn when compared to all other groups tested (Two-way ANNOVA *F* (1, 33) = 13.68, *p* < 0.05; Tukey’s multiple comparison, *p* = 0.0001) (Fig. 3a-d, i). Immunofluorescence co-staining with Iba1 and MHCII showed that the cells expressing MHCII in heterozygous nude rats injected with AAV9-αsyn are microglia or monocytes co-expressing Iba1 (Fig. 3k). Although we observed a few cells staining MHCII alone, more than 95% of the cells expressing MHCII co-stained with Iba1. These results indicate that that although microglia in both genotypes respond to the presence of α-synuclein, infiltration of T cells is necessary for microglia to upregulate MHCII leading to dopamine neuron loss in the SNpc.

### Equivalent expression of α-synuclein in both nude and heterozygous rats

**Fig. 4:**
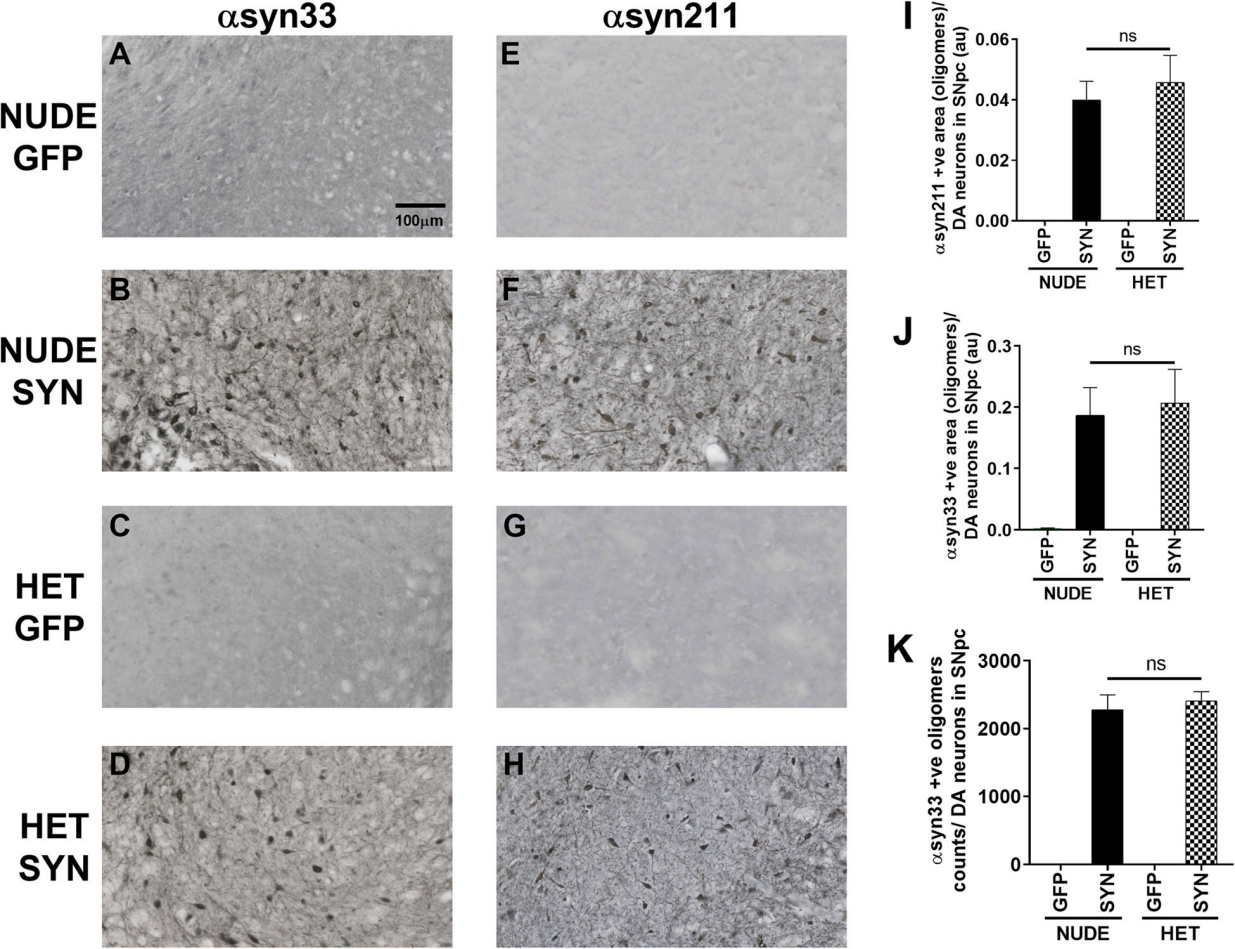
**a**-**d** Representative photomicrographs of α-syn33 (marker for oligomeric α-synuclein) staining. **e**-**h** Representative photomicrographs of α-sy211 (marker for oligomeric α-synuclein) staining. **a**, **e** T cell deficient (nude) rats injected with AAV9-GFP (*n* = 10); **b**, **f** T cell deficient rats injected with AAV9-human α-synuclein (αsyn) (*n* = 10); **c**, **g** T cell competent (heterozygous nude) rats injected with AAV9-GFP (*n* = 10); **d**, **h** T cell competent rats injected with AAV9-human α-synuclein (*n* = 10). **i** Bar graph shows the expression level of α-synuclein oligomers by staining with anti-αsyn211 antibody in SNpc region when examined 2-month post-surgery. Both nude rats and heterozygous nude rats injected with AAV9- α-syn showed similar range of α-synuclein oligomer expression with respect to the dopamine neurons in the SNpc region. **j** Bar graph shows the area units of the α-syn oligomer expression when stained with the α-syn33 antibody (donation from Dr. Rakez Kayed [28]). Like the α-syn211 staining, no significant difference was observed in the expression level of α-syn oligomers in the two groups treated with AAV9-α-syn with respect to the dopamine neurons in the SNpc region. **k** Bar graph shows the number of α-syn oligomers (stereology counting) in the SNpc region with respect to the dopamine neurons. Like the NearCYTE software comparison, stereology counting of α-syn oligomers did not show any significant difference between both groups treated with AAV9-α-syn. The bars in the graph represent mean ± standard error of the mean

To ensure that there were no differences in the amount of α-syn expression between groups we stained with anti-α-syn211 (Fig. 4a-d) and syn33 antibody (Fig. 4e-h), both are markers for oligomeric synuclein in the brain. The α-syn211 antibody binds to phosphorylated S129 on human α-syn, an important post translational modification required for the oligomer formation [32]. Our results indicate that both nude and heterozygous nude rats show the same level of α-syn211 staining at 60 days when normalized to TH expression (Fig. 4i). We additionally stained for oligomeric α-syn expression using an antibody (syn33-(29)) that stains for dimers and higher molecular weight aggregates. Our results show equal expression of α-syn oligomers when normalized to the number of DA neurons using both stereology counting and the ROI area of staining methods in both the groups (Fig. 4 j-k). These results further support that dopamine neuronal loss in the SNpc of the heterozygous nude rats is due to the interaction between T cells and microglia to upregulate MHCII and not due to differences in transgene expression.

### CD4 and CD8 T cells are present in SNpc with α-synuclein expression

**Fig. 5:**
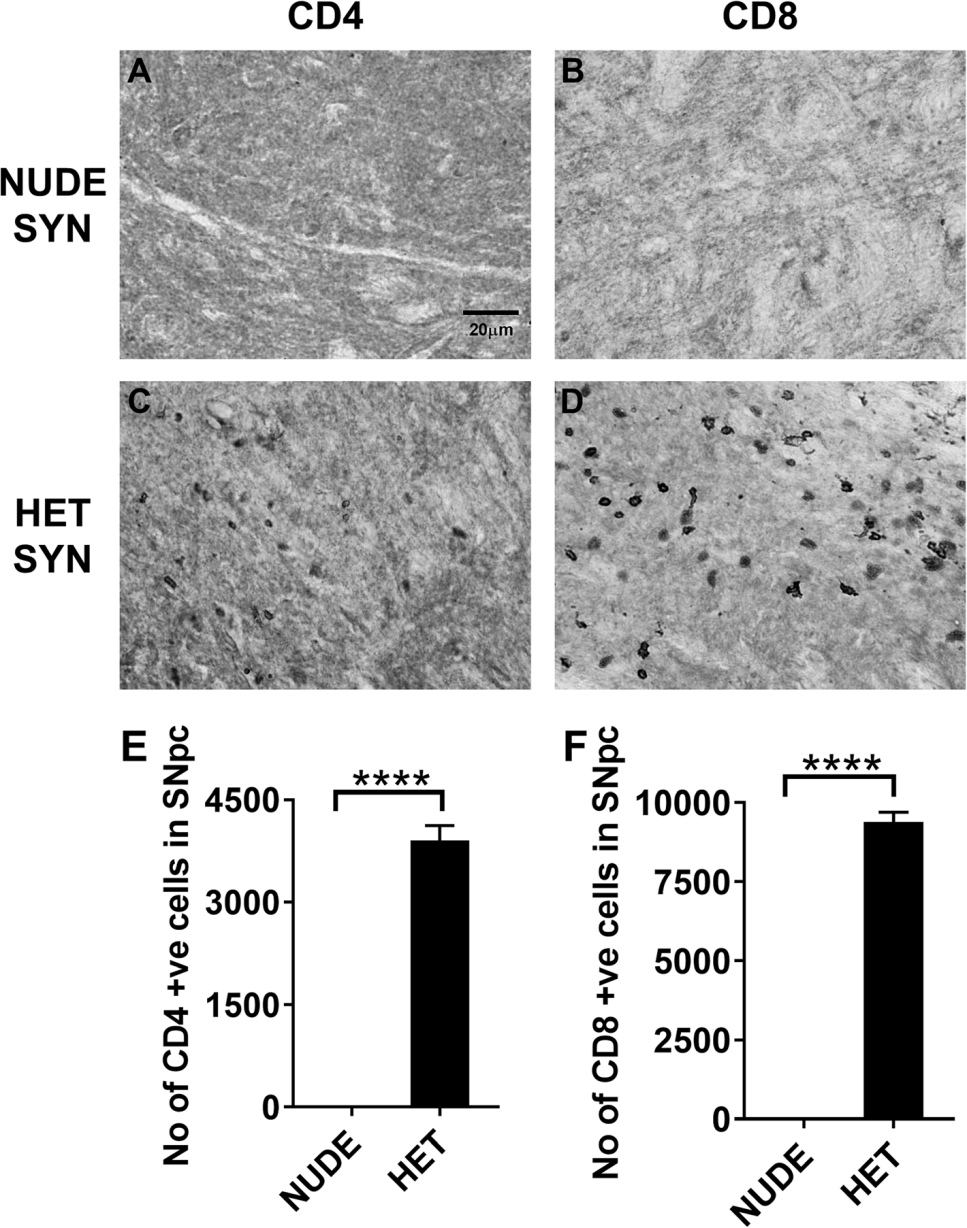
**a**, **c** Representative photomicrographs of CD4 T cell staining. **b**, **d** Representative photomicrographs of CD8 T cell staining. **a**, **b** T cell deficient (nude) rats injected with AAV9-human α-synuclein (αsyn) (*n* = 5); **c**, **d** T cell competent (heterozygous nude) rats injected with AAV9-human α-synuclein (*n* = 5). **e** Bar graph shows the number of CD4 T cells in the injected side of the SNpc region. A significant difference in the number of CD4 T cells was observed between nude and heterozygous nude rats injected with AAV9-α-syn. **f** Bar graph shows the number of CD8 T cells in the injected side of the SNpc region. A significant difference was observed in the number of CD8 T cells between nude and heterozygous nude rats injected with AAV9-αsyn (One way ANNOVA *p* < 0.0001; *F* (3, 14) = 426.6; post hoc analysis: Tukey’s multiple comparison test, *p* < 0.0001). The bars in the graph represent mean ± standard error of the mean

Since microglia upregulate MHCII in heterozygous nude rats injected with AAV9-α-syn, we stained for specific subtypes of T cells in SNpc (Fig. 5a-d). The heterozygous nude rats showed presence of both CD4 and CD8 T cells while the nude rats did not have any T cells present in SNpc with oligomeric α-syn expression (One-way ANNOVA *F* (3, 14) = 426.6, *p* < 0.05; Tukey’s multiple comparison, *p* < 0.0001) (Fig. 5e, f). In a preliminary study, we have shown a significant infiltration of both CD4 and CD8 T cells into the SNpc 30 days post injection in F344 rats (with complete T cell population) injected with AAV9-α-syn when compared with AAV9-GFP injected rats (Supplemental Figure 3A-D). The F344 rats injected with AAV9-α-syn showed a significant increase (One-way ANNOVA *F* (3, 10) = 120.7, *p* < 0.05; Tukey’s multiple comparison, *p* < 0.0001) in the number of CD4 and CD8 positive T cells when compared to the GFP injected controls (Supplemental Figure 3E). These results indicate that T cells infiltrate into SN at a time that coincides with microglial upregulation of MHCII in response to oligomeric α-syn expression, supporting that T cells may be involved in microglial upregulation of MHCII.

### CD4, CD8 T cell infiltration, microglial MHCII expression coincides with dopamine neuron loss

**Fig. 6:**
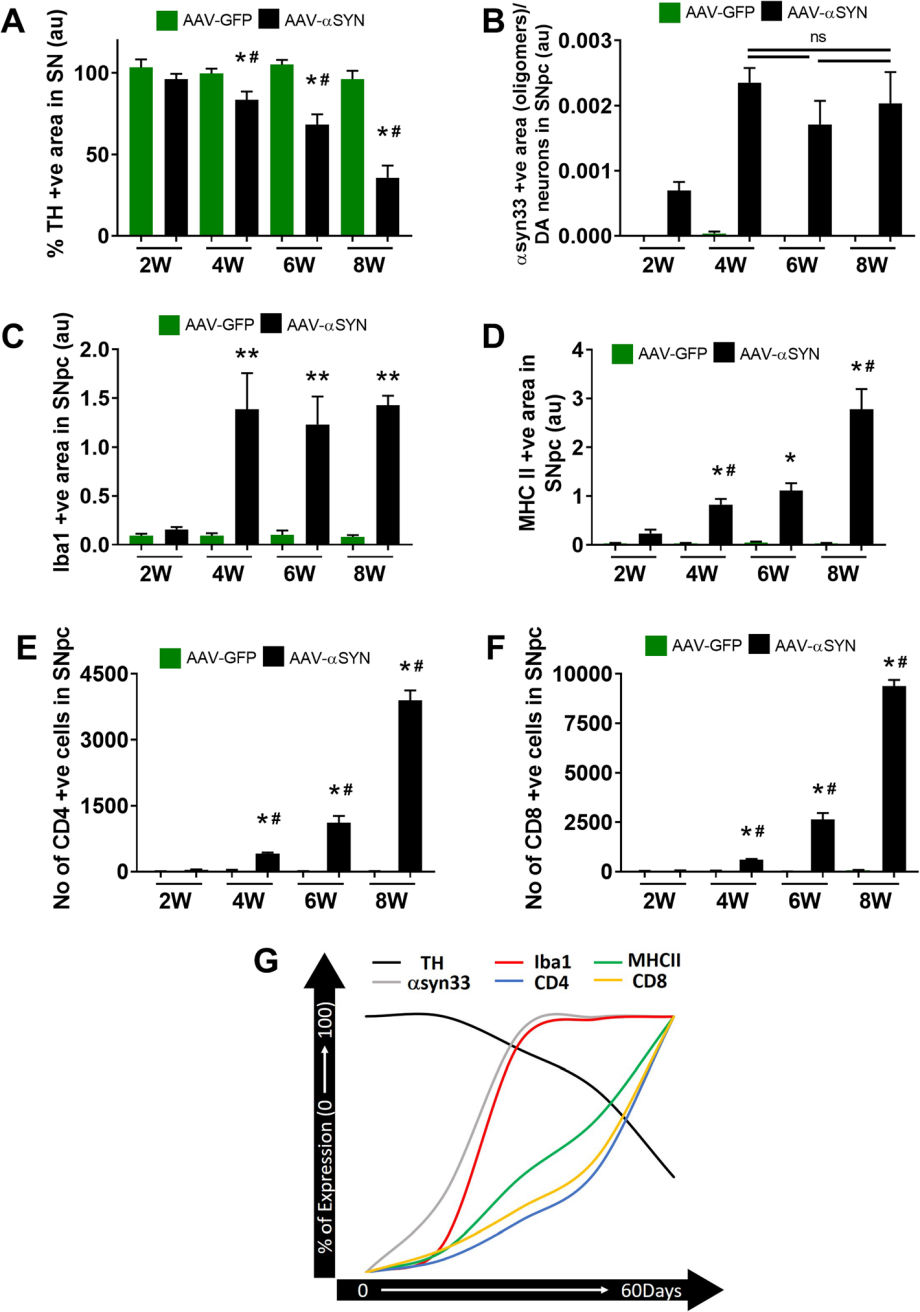
**a** Bar graph shows the percentage area of TH+ positive staining in SNpc by comparing the injected ipsilateral to the untreated contralateral side in heterozygous nude rats at four timepoints (2 weeks, 4 weeks, 6 weeks, 8 weeks) injected with AAV9-GFP (*n* = 3) or AAV9-α-syn (*n* = 5). A significant difference (Two-way ANOVA *p* < 0.05; treatment: *F* (1, 24) = 243.8, *p* < 0.01; timepoint: *F* (3, 24) = 55.62, *p* < 0.01; post hoc analysis: Tukey’s multiple comparison test) of percentage positive cells was observed between the heterozygous nude rats injected with αsyn when compared to GFP injected controls at 4 (30 days), 6 (45 days), and 8 (60 days) weeks timepoint. A progressive loss of dopamine neurons was observed in heterozygous nude rats injected with AAV9-α-syn when compared at 4, 6, and 8 weeks timepoint. **b** Bar graph shows the area units of the α-syn oligomer expression by staining with the α-syn33 antibody (donation from Dr. Rakez Kayed [29]) of the heterozygous nude rats injected with AAV9-GFP (*n* = 3) or AAV9-α-syn (*n* = 5) at four different timepoints. At 2 weeks (14 days) α-syn expression begins to be observed and then increases further at 4 weeks (30 days). No significant difference was observed in the expression level of α-syn oligomers in the heterozygous nude rats treated with AAV9-α-syn normalized to the dopamine neurons in the SNpc region at 4, 6, and 8 weeks timepoint. **c** Bar graph shows the area units for Iba1 staining for microglia in heterozygous nude rats injected with either AAV9-GFP (*n* = 3) or AAV9-α-syn (*n* = 5) in the SNpc region at four different timepoint. Heterozygous nude rats injected with AAV9-α-syn showed increased number of Iba1 cells in SNpc region when compared to GFP injected controls at 4, 6, and 8 weeks (Two-way ANOVA *p* < 0.0001; treatment: *F* (1, 24) = 36.01, *p* < 0.0001; post hoc analysis: Tukey’s multiple comparison test). No significant difference was observed between heterozygous nude rats injected with AAV9-αsyn at 4, 6, and 8 weeks timepoint. **d** Bar graph shows the area units for MHC II staining for activated microglia in heterozygous nude rats injected with either AAV9-GFP (*n* = 3) or AAV9-α-syn (*n* = 5) at four different timepoints. The microglia express MHCII only in the heterozygous nude rats injected with AAV9-α-syn in SNpc region gradually increasing from 4 weeks to 8 weeks (Two-way ANOVA *p* < 0.001; treatment: *F* (1, 24) = 60.63, *p* < 0.0001; timepoint: *F* (3, 24) = 12.43, *p* < 0.0001; post hoc analysis: Tukey’s multiple comparison test). The MHCII positive microglia was observed higher in 8 weeks timepoint when compared to the 6 and 4 weeks coinciding with dopamine neuron loss in SNpc. **e** Bar graph shows the number of CD4 T cells in the injected side of the SNpc region in heterozygous nude rats at four different timepoints. A significant difference in the number of CD4 T cells was observed in heterozygous nude rats injected with AAV9-αsyn when compared to GFP injected controls at 4, 6, and 8 weeks timepoint (Two-way ANOVA *p* < 0.001; treatment: *F* (1, 23) = 205, *p* < 0.0001; timepoint: *F* (3, 23) = 86.33, *p* < 0.0001; post hoc analysis: Tukey’s multiple comparison test). Also, a gradual increase in the number of CD4 T cells was observed in heterozygous nude rats injected with AAV9-α-syn as time increases. **f** Bar graph shows the number of CD8 T cells in the injected side of the SNpc region in heterozygous nude rats at four different timepoints. A significant difference in the number of CD8 T cells was observed in heterozygous nude rats injected with AAV9-αsyn when compared to GFP-injected controls at 4, 6, and 8 weeks timepoint (two-way ANOVA *p* < 0.001; treatment: *F* (1, 23) = 396.7, *p* < 0.0001; timepoint: *F* (3, 23) = 190.7, *p* < 0.0001; post hoc analysis: Tukey’s multiple comparison test). Also, a gradual increase in the number of CD8 T cells was observed in heterozygous nude rats injected with AAV9-α-syn as time increases. *p < 0.05, **p < 0.001 when compared to GFP injected controls at a given timepoint; #*p* < 0.05 when compared to α-syn injected heterozygous nude rats at the previous timepoint. The bars in the graph represent mean ± standard error of the mean. **g** A representative figure explaining the timeline of dopamine neuron loss (TH) coinciding with microglial MHCII activation and CD4 and CD8 T cells infiltration along with Iba1 microglia and α-syn33 oligomer expression attaining stable expression after 4 weeks of AAV9-α-syn injection. The timeline for each immunohistochemistry staining represented here is percentage of expression attaining 100% either at 0 or 8 weeks (60 days)

We quantified dopamine neuron loss, microglial MHCII expression, αsyn oligomers, CD4, and CD8 T cells in nude and heterozygous nude rats at 60 (8 weeks) days timepoint. To understand the relationship between the timeline of neuron loss, microglial activation, and T cell infiltration post α-syn injection, we analyzed heterozygous nude rats injected with either AAV9-GFP or AAV9-α-syn at 2-week intervals. A progressive loss of dopamine neurons was observed in heterozygous nude rats injected with AAV9-α-syn with progressive loss over time (Two-way ANOVA *p* < 0.05; treatment: *F* (1, 24) = 243.8, *p* < 0.01; timepoint: *F* (3, 24) = 55.62, *p* < 0.01; post hoc analysis: Tukey’s multiple comparison test) (Fig. 6a). Similarly, a gradual increase in the number of activated microglia expressing MHCII was observed with time coinciding with neuron loss (Two-way ANOVA *p* < 0.001; treatment: *F* (1, 24) = 60.63, *p* < 0.0001; timepoint: *F* (3, 24) = 12.43, *p* < 0.0001; post hoc analysis: Tukey’s multiple comparison test) (Fig. 6d). However, the expression of Iba1 and α-syn33 oligomers in SNpc (normalized to TH expression) increased at 15 (2 weeks) and 30 (4 weeks) days and remained stable over time up to 60 (8 weeks) days (Two-way ANOVA *p* < 0.0001; treatment: *F* (1, 24) = 36.01, *p* < 0.0001; post hoc analysis: Tukey’s multiple comparison test) (Fig. 6b, c). Concurring with microglial MHCII expression, the number of both CD4 and CD8 T cells also increased progressively with time in SNpc of heterozygous rats expressing αsyn (Fig. 6e, f). In Fig. 6g, we show the relationship between all of these events. The changes that most closely coincide with loss of TH staining are the expression of MHCII, CD4, and CD8 T cell. Thus, although microglia respond to the presence of α-synuclein by showing upregulation of Iba1 and changes in morphology (as shown in Fig. 3), this remains stable over time, the loss of TH more closely follows the expression of MHCII and T cell infiltration.

### LPS and TNF-α both stimulate microglia ex vivo

**Fig. 7:**
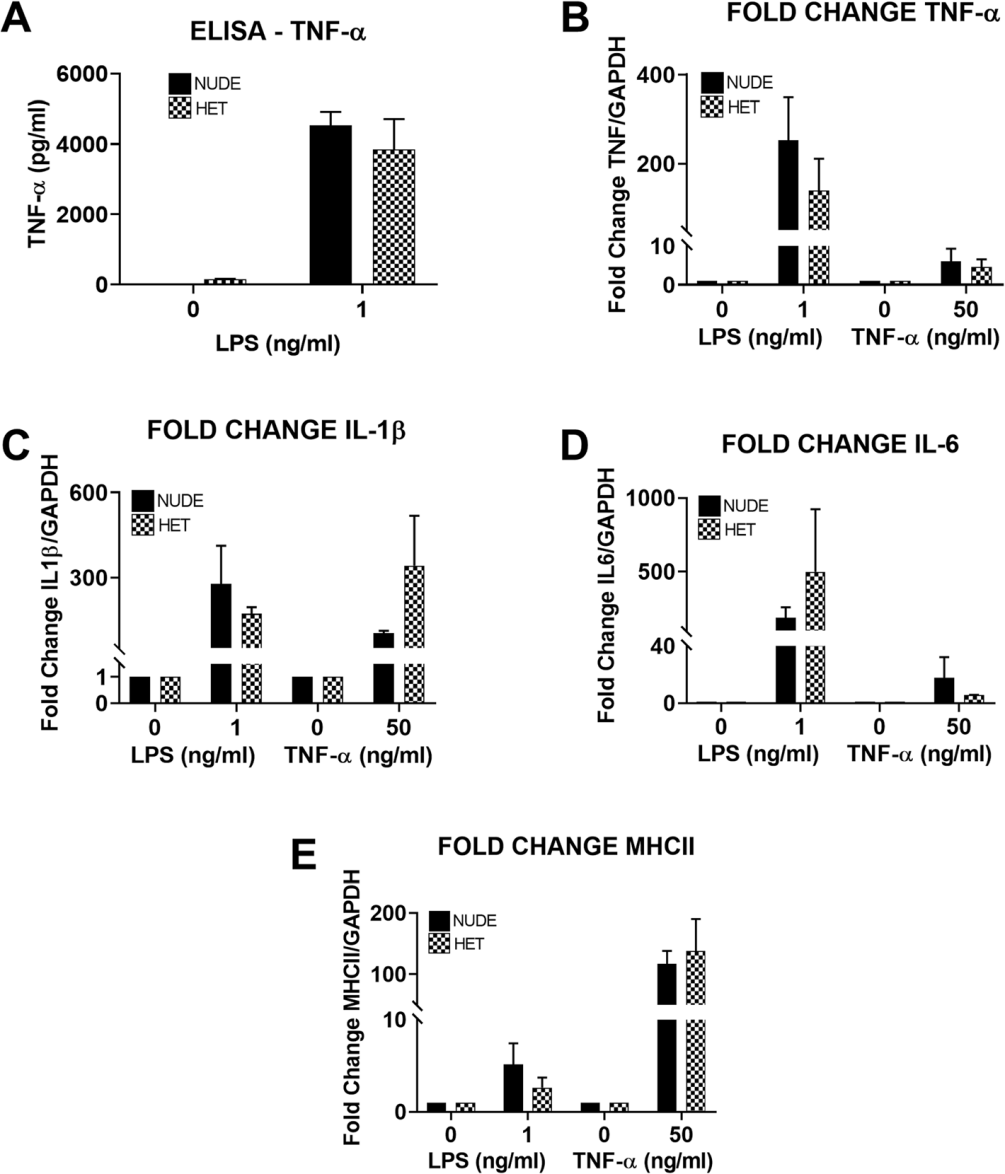
**a** Bar graph showing the concentration of TNF-α (pg/ml) secreted in the media isolated from the primary microglia cultures from nude (*n* = 2; experimental replicates: *n* = 3) and heterozygous nude rats (*n* = 2; experimental replicates: *n* = 3) incubated with LPS (1 ng/ml) for 3 h. Primary microglia isolated from both nude rats and heterozygous nude rats responded to LPS by secreting pro-inflammatory cytokine TNF-α into the media. **b**-**e** Bar graphs represent the fold change of the TNF, IL1β, IL6, and MHCII RNA expression in the primary microglial cells (nude and heterozygous nude rats) when treated with LPS (1 ng/ml) and TNF-α (50 ng/ml). Both primary microglial cultures showed increased RNA expression of inflammatory cytokines (Two-way ANNOVA, *p* < 0.01; post hoc analysis: Tukey’s multiple comparison) when treated with LPS and TNF-α indicating that primary microglia is activated in both nude and heterozygous nude rats in a similar manner to various stimuli when compared to untreated controls. The bars in the graph represent mean ± standard error of the mean

Our results show that T cells infiltration coincides with the expression of MHCII in microglia in response to expression of α-syn in the SNpc. In order to test if the microglia from nude rats show normal expected pro-inflammatory responses to other stimuli such as LPS and TNF-α, we isolated primary microglial cells from nude and heterozygous nude rats and exposed them to LPS (1 ng/ml) or TNF-α (50 ng/ml) in vitro. From both genotypes, we isolated around 2 million microglia per brain showing no differences in absolute numbers of microglia in untreated rats. Following LPS exposure, the amount of TNF-α secreted in the media was tested using ELISA (Fig. 7a). Microglial cells isolated from both nude and heterozygous nude rats secreted equivalent amounts of TNF-α in response to LPS treatment, demonstrating that the lack of T cells in the nude rats does not alter the microglial cell’s ability to respond to the pro-inflammatory stimulation by LPS. Furthermore, extraction of RNA from isolated microglial cells exposed to either LPS or TNF-α demonstrated that induction of the pro-inflammatory genes, TNF-α, MHCII, IL1β, and IL6 using qRT-PCR, were similar in microglia cells from both nude and heterozygous nude rats (Fig. 7b-e). These results suggest that microglia from nude and heterozygous nude rats are able to respond equally to various pro-inflammatory stimuli and further underscore that the lack of increased MHCII expression in response to α-synuclein in the athymic nude rats is due to the absence of T cells and not due to a reduction in response to other pro-inflammatory activators. Further work is needed to explore the specific subtype of T cell and its role in the stimulation of the microglial response.

## Discussion

Clinical symptoms of PD includes tremor, postural instability, slowness of movement, and rigidity [2] characterized using behavioral test such as locomotor activity, rotarod, and forepaw cylinder test in rodent models. We have previously shown that AAV9-induced expression of α-syn results in dopamine neuronal loss in SNpc and is accompanied by forelimb akinesia as assessed by the cylinder test [26]. In our study, we observed a similar forelimb akinesia in T cell competent rat expressing α-syn but not in T cell deficient rats. Symptom onset in PD happens almost after 50% loss of DA neurons in SNpc affecting nigrostriatal pathway suggesting that T cell deficient rats may not have undergone extensive DA neuron loss in SNpc at the timepoint we analyzed. Though, the underlying cause for neurodegeneration and accumulation of α-syn in PD remains unclear, there is increasing evidence linking environmental factors, genetics, and failure in the ubiquitin-proteasome pathway in clearing out the damaged proteins [33]. Pro-inflammatory changes in microglia, resident innate immune cells of the CNS, resulting from α-syn accumulation has been suggested as a likely cause of the neuronal loss in SNpc [11, 16, 34–37]. Microglia survey the brain environment for any damage to neurons, presence of any foreign antigens, and synaptic functionality. In their resting state, microglia survey by cell-cell interactions and fluid endocytosis (pinocytosis) of the extracellular matrix [38]. Encountering a foreign antigen or small pathogen, microglia change their number, morphology, and cell surface receptors and become activated. Depending on the exact manifestation of microglial response to the microenvironment neurodegeneration occurs in a diseased brain. Pro-inflammatory microglia are responsible for cascades of cytokines and chemokines; however, the balance in this response can have either beneficial or detrimental effects on the site of damage. In our study, we found extensive microglial changes including increased cell body diameter, number of cells in the SNpc, and upregulation of MHCII (Fig. 3) along with dopamine neuron loss (Fig. 2) in heterozygous nude rats injected with AAV9-α-syn corroborating previous studies. The role of microglia has been increasingly explored in pathogenesis of Parkinson’s disease; however, the role of adaptive immune system such as T cells and B cells is still being explored [39, 40].

Post-mortem human PD brains have shown the presence of both CD4+ (helper) and CD8+ (cytotoxic) T cells suggesting that ongoing neurodegeneration may recruit them to the area of damage [20]. The role of T cells in PD has been increasingly explored in neurotoxic symptomatic models like MPTP, 6-hydroxydopamine. MPTP toxicity is reduced in T cell deficient mice suggesting a neurotoxic role for T cells in PD [20, 23, 41, 42]. Reports have also shown that in postmortem human PD brains, microglia activated by α-syn accumulation secrete cytokines activating dopamine neurons in SNpc to express MHC class I on the cell surface and in turn, CD8+ T cells kill these neurons that express the right combination of α-syn peptide and MHC class I receptor [43]. Nevertheless, the role of T cells and T cell subtypes in an α-syn-mediated neurodegeneration is still not fully understood. In this study, we have demonstrated that T cells are necessary for the upregulation of MHCII in microglia and that this appears to be an important step in dopamine neuronal loss in SNpc in response to α-synuclein. The microglia in both nude and heterozygous rats do show a response to α-synuclein as demonstrated by clear changes in morphology in both genotypes. However, only in the heterozygous rats was the response to α-synuclein more pronounced, both when measured with Iba1 and the expression of MHCII (Fig. 3). We use a PD model that overexpresses human wild-type α-syn using adeno-associated virus serotype 9 in SNpc, which closely mimics many aspects of both genetic and sporadic disease pathology in humans. We demonstrate that with equal levels of α-syn accumulation within the SNpc, dopaminergic neuron cell death in nude rats (T cell deficient) and heterozygous nude rats (T cell competent) is markedly different. The amount of cell loss was significantly higher in the heterozygous nude rats that are T cell competent and we also observe upregulation of MHCII in microglia. Furthermore, a comparison to F344 rats which have a fully competent adaptive immune system, injected with equivalent amount of AAV9-α-syn showed even greater and faster loss of DA neurons. This suggests that there could be a T cell dose dependent cell loss, although we cannot fully rule out that this effect might have been related to background strain differences between the athymic nude and F344 rats.

Our results indicate that presence of α-syn alone may not be sufficient to cause significant cell death of dopamine neurons in SNpc and that T cells are required for this process, at least in the time frame studied here. It is possible that a longer exposure to α-synuclein in the absence of T cells would have resulted in additional loss of DA neurons and that the presence of T cells simply accelerates the process by upregulation of MHC class II in microglia. α-synuclein expression alters microglia in both athymic nude and heterozygous nude rats but presence of T cells increases microglial expression Iba1 as well as upregulation of MHCII in the SNpc, indicating that the presence of T cells is likely involved in the upregulation of MHC class II in microglia (monocyte) that may be necessary, or at least accelerate, dopaminergic cell loss.

The major hallmark difference between nude rats and heterozygous nude rats is the absence and presence of mature T cells (Supplementary Figure 1). Although heterozygous nude rats are immune competent, when comparing to F344 rat they carry a lower population of T cells (Supplemental Figure 1). Genotypic difference between nude and heterozygous nude rats did not affect the viral expression of α-syn as we observed similar expression of α-syn oligomers in SNpc by both nude and heterozygous nude rats. Supporting previous studies in human post-mortem PD brains [20], we also observed CD4+ and CD8+ T cells in SNpc of both heterozygous nude rats and F344 rats but not in nude rats (Fig. 5, Supplementary Figure 3). The infiltration of T cells coincided with microglial MHCII expression indicating a strong connection between microglial MHCII expression, CD4, CD8 T cell infiltration, and DA neuron loss. Studying the timeline of dopamine neuron loss in T cell competent rats revealed that although α-synuclein expression and the response of microglia to upregulate Iba1 occurs by 30 (4 weeks) days, they both remain stable until 60 (8 weeks) days. Conversely, there was a gradual increase in microglial expression of MHCII, CD4, and CD8 T cell infiltration that mirrored the progressive decline in dopamine neurons in SNpc (Fig. 6). However, whether the initiation factor for dopamine neuron loss is microgliosis or T cell infiltration is unknown. Microglia from two genetically different rats used in this study did not have any discrepancy in terms of inflammatory response to various stimuli. The pro-inflammatory response of microglia to LPS and TNF-α was similar in both nude and heterozygous nude rats. The presence or absence of T cells did not affect the microglial response to these stimuli in treatment ex vivo (Fig. 7). This further underscores that the absence of DA cell loss observed in the nude rats is related to the absence of T cells and not a general difference in microglial function. Taken together, these results suggest that the interaction of T cells with microglia involving upregulation of MHC class II is necessary for dopamine neuronal loss in SNpc and the progressive decline in dopamine neurons is concurrent with increase in microglial upregulation of MHCII and T cell infiltration. This is in agreement with previous studies demonstrating that in MHCII deficient rodents, α-synuclein is less toxic [23]. Our findings demonstrate a role for T cells in this interaction.

From our study in this AAV-α-syn model of PD, T cells are required for microglial upregulation of MHCII and subsequent loss of DA neurons in the SNpc. This indicates that both the presence of T cells and microglial upregulation of MHCII are important for loss of dopamine neurons in the SNpc. This study examines the contribution of T cells by using a model that lacks all T cells including both cytotoxic (CD8+) and helper (CD4+) T cells; therefore, the role of the specific subtypes of T cells in neurodegeneration in α-syn model of PD needs to be further explored. In a human autologous iPSC-based model and an MPTP model of PD, T helper (Th) 1 and Th17, the pro-inflammatory phenotypes of CD4+ T cells, are shown to be involved in the degeneration of neurons [42, 44]. However, the anti-inflammatory phenotypes of CD4+ T cells such as Th2 and Treg are shown to be neuroprotective, but are not elevated in SN in an MPTP model of PD. Administration of Treg to MPTP mice led to robust protection against dopamine neuron loss in the SN [42]. The exact role of the T helper and Treg cells in the AAV-α-syn model of PD needs to be explored further. Specific subtypes of T cells are found to be key players in diseases like multiple sclerosis and rheumatoid arthritis [45]. Drug candidates targeting against Th17/IL17 pathway have been approved for various inflammatory disorders such as psoriasis, rheumatoid arthritis, multiple sclerosis, and Crohn’s disease [46]. Understanding their association in PD pathogenesis will help to revise our knowledge on how dopamine neuronal loss in SNpc might be driven by systemic inflammation caused by T cells. Targeting specific subtype of T cells entry to CNS might be a better therapeutic model in the treatment of PD patients.

## Conclusions

In summary, we clearly demonstrate that the AAV9 α-syn-mediated loss of DA neurons in the SNpc is dependent on the presence of T cells and their interaction with microglia to upregulate MHCII. There was no significant loss of DA neurons in the athymic nude rats unlike the heterozygous nude rats which had significant DA neuron loss, despite the presence of similar amounts of oligomeric α-synuclein. This emphasizes that neuroinflammation, T cell infiltration in SNpc, and microglial upregulation of MHCII is a key component to cell death in this model of PD pathology.

## Supplementary information

**Additional file 1.** Supplemental Figure 1. (A – B) Representative photomicrographs of CD4 T cell staining of the spleen (Periarterial lymphoid sheath (PALS) region). (D – F) Representative photomicrographs of CD8 T cell staining of the spleen (PALS region). A, D – T cell deficient nude rat spleen (n = 3); B, E – T cell competent heterozygous nude rat spleen (n = 5); C, F – T cell competent Fischer 344 rat spleen (n = 3). (G) Bar graph shows the number of CD4 and CD8 T cells in one PALS region of the spleen. A significant difference was observed between nude rats and T cell competent (heterozygous nude and F344 wild-type rats) rats, also between heterozygous nude and F344 wild-type rats for both CD4 and CD8 T cells staining (Two-way ANNOVA, p < 0.01; F(1, 16) = 15.54; Post-Hoc analysis: Sidak’s multiple comparison test, p < 0.0001). The bars in the graph represent mean ± standard error of the mean.

**Additional file 2.** Supplemental Figure 2. Bar graph shows the number of positive cells in SNpc stained with TH in the ipsilateral side injected with AAV9-GFP (n = 5) or AAV9-αsyn (n = 6). A significant difference (Two-way ANOVA p < 0.05; Treatment: F (1, 18) = 15.01, p < 0.01; Genetic Background: F (1, 18) = 5.266, p < 0.05; Post-Hoc analysis: Tukey’s multiple comparison test) of percentage positive cells was observed between the heterozygous nude rats injected with αsyn when compared to GFP injected controls. No significant difference was observed between the nude rats injected with αsyn compared to GFP injected controls.

**Additional file 3.** Supplemental Figure 3. (A-B) Representative photomicrographs of CD4 T cell staining of Fisher 344 rats (n = 5). (C-D) Representative photomicrographs of CD8 T cell staining of Fisher 344 rats (n = 5). A, C – F344 rats injected with AAV9-GFP; B, D – F344 rats injected with AAV9-α-syn. (E) Bar graph shows the number of CD4 and CD8 T cell (stereology counted) in the SNpc region of F344 rats. The F344 rats injected with AAV9-α-syn showed an increased number of both CD4 and CD8 T cells in the SNpc region when compared to the GFP injected controls (One-way ANNOVA, p < 0.05; F(3, 10) = 120.7; Post Hoc analysis: Tukey’s multiple comparison test, p < 0.0001).

## Data Availability

The datasets generated and/or analyzed in this study are available from the corresponding author upon request.

## Abbreviations

AAV: Adeno-associated virus
α-syn: α-synuclein
CD: Cluster of differentiation
CNS: Central nervous system
DA: Dopamine
F344: Fischer 344
GFP: Green fluorescent protein
IL: Interleukin
L-DOPA: L-3, 4-dihydroxyphenylalanine
LPS: Lipopolysaccharide
MHC II: Major histocompatibility complex II
MPTP: 1-methyl-4-phenyl-1,2,3,6-tetrahydropyridine
PD: Parkinson’s disease
RT-PCR: Reverse transcription polymerase chain reaction
SNpc: Substantia nigra pars compacta
TNF: Tumor necrosis factor
TH: Tyrosine hydroxylase
