# Supplementary figures and images for: T cell infiltration and upregulation of MHCII in microglia leads to accelerated neuronal loss in an α-synuclein rat model of Parkinson’s disease
Source: J Neuroinflammation. 2020 Aug 15;17:242. doi: 10.1186/s12974-020-01911-4 (PMC7429710; doi:10.1186/s12974-020-01911-4)

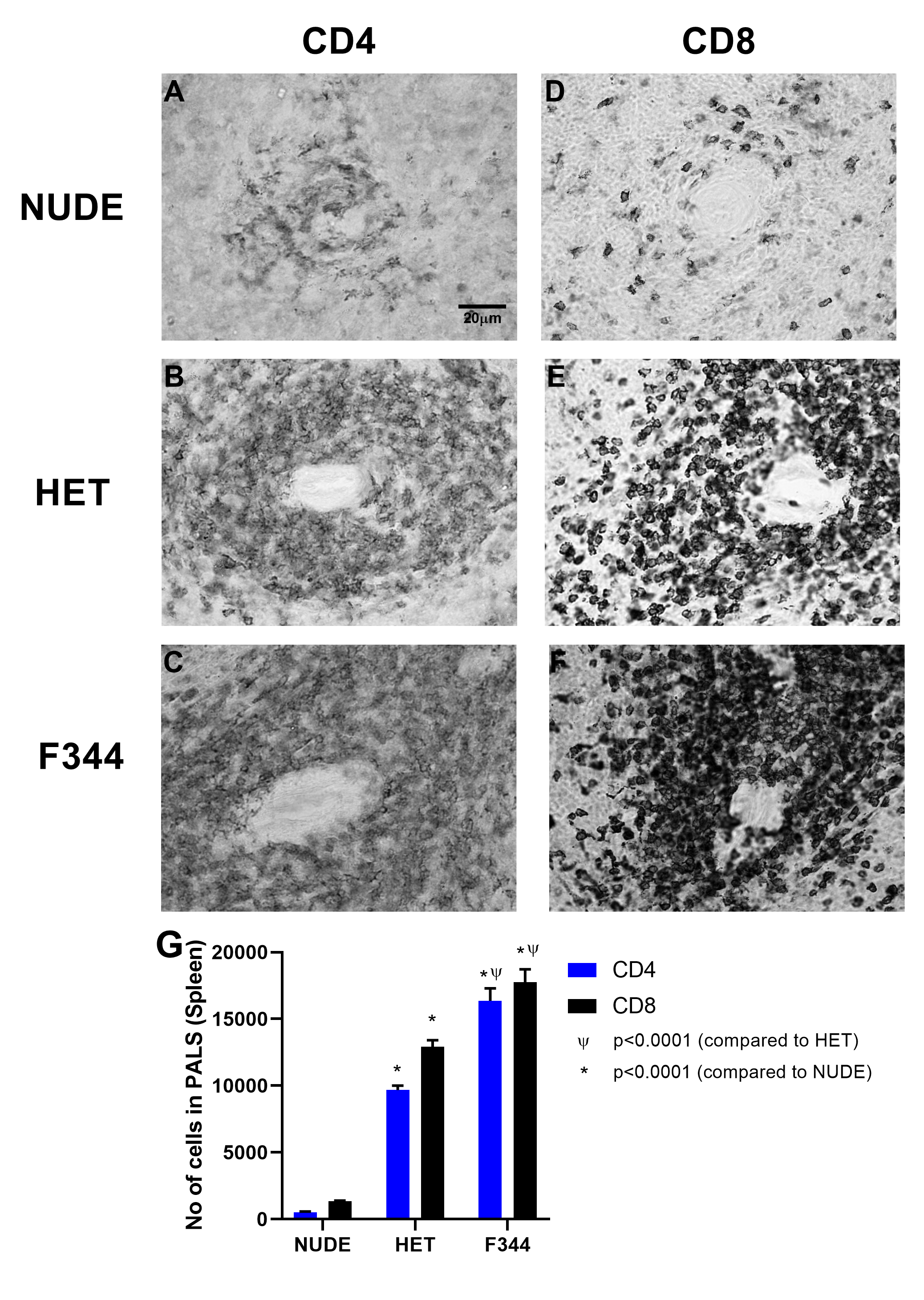

Supplement: Supplementary file 1 — Additional file 1. Supplemental Figure 1. (A – B) Representative photomicrographs of CD4 T cell staining of the spleen (Periarterial lymphoid sheath (PALS) region). (D – F) Representative photomicrographs of CD8 T cell staining of the spleen (PALS region). A, D – T cell deficient nude rat spleen (n = 3); B, E – T cell competent heterozygous nude rat spleen (n = 5); C, F – T cell competent Fischer 344 rat spleen (n = 3). (G) Bar graph shows the number of CD4 and CD8 T cells in one PALS region of the spleen. A significant difference was observed between nude rats and T cell competent (heterozygous nude and F344 wild-type rats) rats, also between heterozygous nude and F344 wild-type rats for both CD4 and CD8 T cells staining (Two-way ANNOVA, p < 0.01; F(1, 16) = 15.54; Post-Hoc analysis: Sidak’s multiple comparison test, p < 0.0001). The bars in the graph represent mean ± standard error of the mean. [file 12974_2020_1911_MOESM1_ESM.tif]

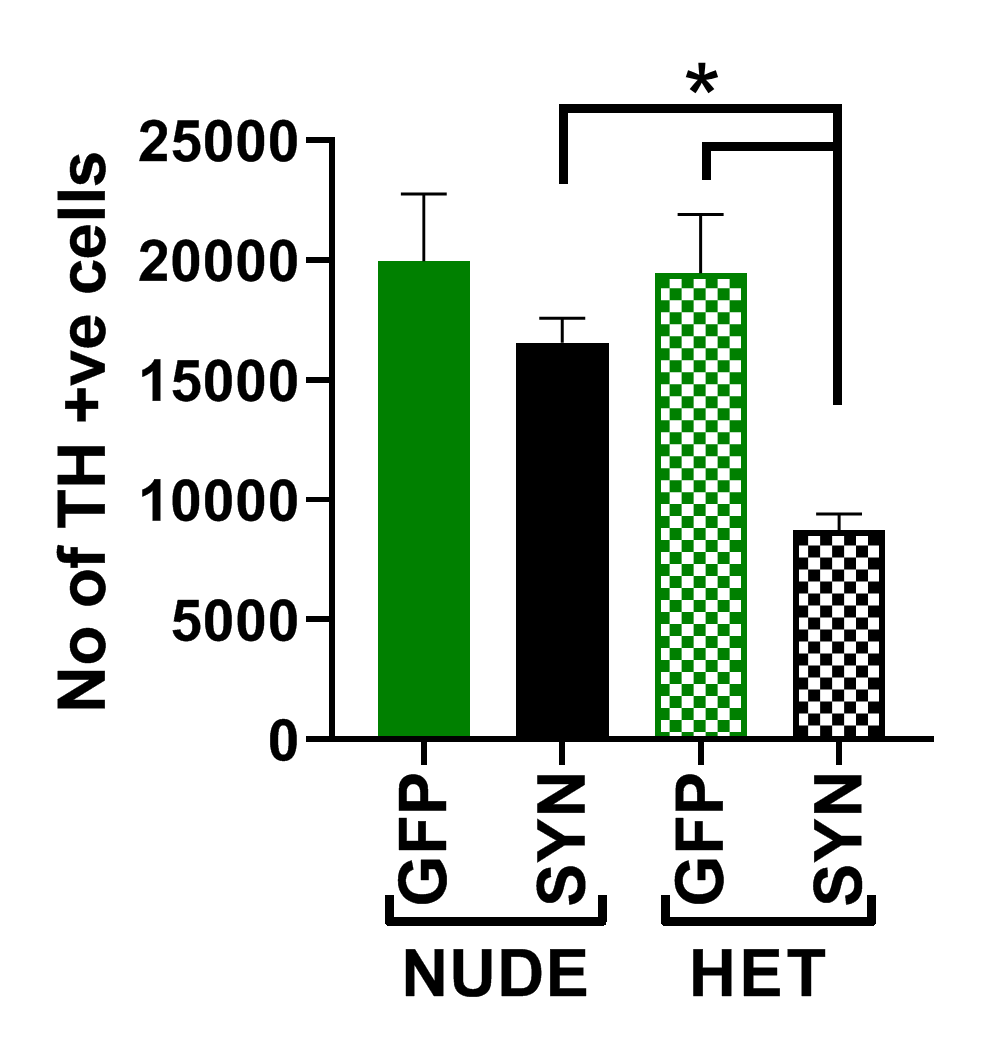

Supplement: Supplementary file 2 — Additional file 2. Supplemental Figure 2. Bar graph shows the number of positive cells in SNpc stained with TH in the ipsilateral side injected with AAV9-GFP (n = 5) or AAV9-αsyn (n = 6). A significant difference (Two-way ANOVA p < 0.05; Treatment: F (1, 18) = 15.01, p < 0.01; Genetic Background: F (1, 18) = 5.266, p < 0.05; Post-Hoc analysis: Tukey’s multiple comparison test) of percentage positive cells was observed between the heterozygous nude rats injected with αsyn when compared to GFP injected controls. No significant difference was observed between the nude rats injected with αsyn compared to GFP injected controls. [file 12974_2020_1911_MOESM2_ESM.tif]

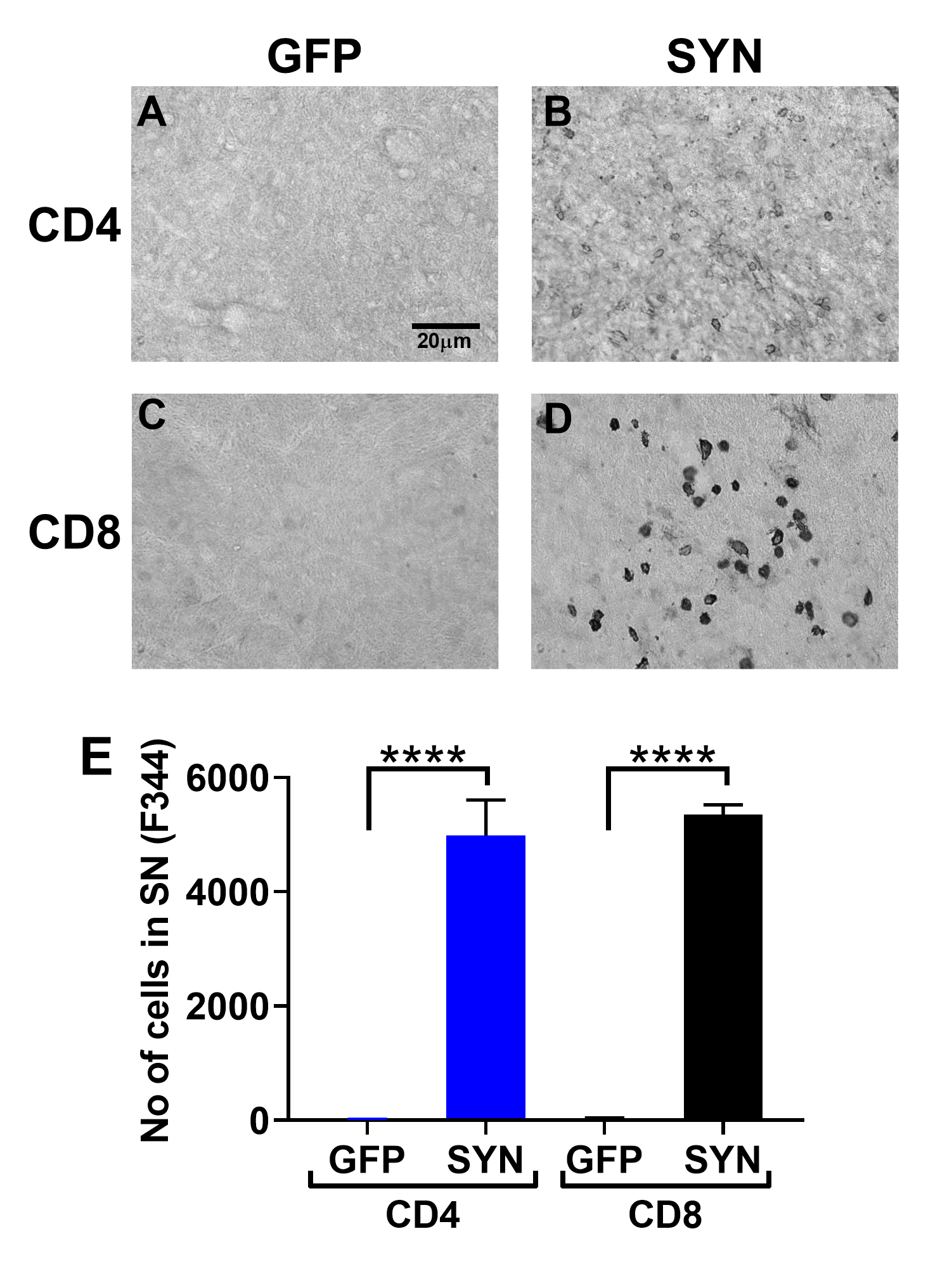

Supplement: Supplementary file 3 — Additional file 3. Supplemental Figure 3. (A-B) Representative photomicrographs of CD4 T cell staining of Fisher 344 rats (n = 5). (C-D) Representative photomicrographs of CD8 T cell staining of Fisher 344 rats (n = 5). A, C – F344 rats injected with AAV9-GFP; B, D – F344 rats injected with AAV9-α-syn. (E) Bar graph shows the number of CD4 and CD8 T cell (stereology counted) in the SNpc region of F344 rats. The F344 rats injected with AAV9-α-syn showed an increased number of both CD4 and CD8 T cells in the SNpc region when compared to the GFP injected controls (One-way ANNOVA, p < 0.05; F(3, 10) = 120.7; Post Hoc analysis: Tukey’s multiple comparison test, p < 0.0001). [file 12974_2020_1911_MOESM3_ESM.tif]
